# Retracted: An Unusual Case of Bilateral Vitreous Haemorrhage following Snake Bite

**DOI:** 10.1155/2018/6235787

**Published:** 2018-01-18

**Authors:** Case Reports in Medicine

Case Reports in Medicine has retracted the article titled “An Unusual Case of Bilateral Vitreous Haemorrhage following Snake Bite” [1]. The article was found to contain a substantial amount of material from the following published articles: “Bhalla, A. et al., “Central retinal artery occlusion: an unusual complication of snakebite,” Journal of Venomous Animals and Toxins Including Tropical Diseases, Botucatu, vol. 10, no. 3, pp. 311–314, 2004, doi: 10.1590/S1678-91992004000300009”; “Jae Seok Kim et al. “Coagulopathy in patients who experience snakebite,” Korean Journal of Internal Medicine, vol. 23, no. 2, pp. 94–99, 2008, doi: 10.3904/kjim.2008.23.2.94”; “Tungpakorn, N., “Unusual visual loss after snakebite,” Journal of Venomous Animals and Toxins Including Tropical Diseases, Botucatu, vol. 16, no. 3, pp. 519–523, 2010, doi: 10.1590/S1678-91992010000300020”; and “Rao, B. M., “A case of bilateral vitreous haemorrhage following snake bite,” Indian Journal of Ophthalmology, vol. 25, no. 1-2, 1977. http://www.ijo.in/article.asp?issn=0301-4738;year=1977;volume=25;issue=2;spage=1;epage=2;aulast=Rao.”

## References

[1] Bhandari V., Lohia M. (2013). An unusual case of bilateral vitreous haemorrhage following snake bite. *Case Reports in Medicine*.

